# Role of serotonin in fish reproduction

**DOI:** 10.3389/fnins.2015.00195

**Published:** 2015-06-05

**Authors:** Parvathy Prasad, Satoshi Ogawa, Ishwar S. Parhar

**Affiliations:** Brain Research Institute, Jeffrey Cheah School of Medicine and Health Sciences, Monash University MalaysiaSelangor, Malaysia

**Keywords:** teleost fish, 5-HT, GnRH, gonadotropins, pituitary, SSRI antidepressants

## Abstract

The neuroendocrine mechanism regulates reproduction through the hypothalamo-pituitary-gonadal (HPG) axis which is evolutionarily conserved in vertebrates. The HPG axis is regulated by a variety of internal as well as external factors. Serotonin, a monoamine neurotransmitter, is involved in a wide range of reproductive functions. In mammals, serotonin regulates sexual behaviors, gonadotropin release and gonadotropin-release hormone (GnRH) secretion. However, the serotonin system in teleost may also play unique role in the control of reproduction as the mechanism of reproductive control in teleosts is not always the same as in the mammalian models. In fish, the serotonin system is also regulated by natural environmental factors as well as chemical substances. In particular, selective serotonin reuptake inhibitors (SSRIs) are commonly detected as pharmaceutical contaminants in the natural environment. Those factors may influence fish reproductive functions via the serotonin system. This review summarizes the functional significance of serotonin in the teleosts reproduction.

## Introduction

Reproduction is a biological process that results in the production of new individual. The nervous and the endocrine system work together (neuroendocrine) to control vertebrate reproduction. The neuroendocrine mechanism regulates reproduction through the hypothalamo-pituitary-gonadal (HPG) axis which is evolutionarily conserved in vertebrates. The hypothalamus is the major site responsible for the production of neuropeptide, gonadotropin-releasing hormone (GnRH) in the brain of vertebrates. In vertebrates, reproductive and sexual functions are mainly controlled by the pulsatile secretion of GnRH from the hypothalamus (Knobil, 1979; Pozor et al., 1991; Dellovade et al., 1998; Bancroft, 2005). GnRH binds to its cognate receptors located on the pituitary gonadotropes to regulate the synthesis and release of gonadotropins: luteinizing hormone (LH) and follicle-stimulating hormone (FSH) (McCann and Ojeda, 1996; McCann et al., 2002). These gonadotropins control gonadal development and maturation, and stimulating steroidogenesis and spermatogenesis in male testes and folliculogenesis and oogenesis in female ovaries (Pierce and Parsons, 1981; Orth, 1984; Bousfield et al., 1994). Furthermore, kisspeptin, the peptide product of *KISS1*/*Kiss1* gene and its cognate receptor (GPR54 = kisspeptin receptor) has been recognized as a potent regulator of GnRH release in mammals (Tena-Sempere, 2006; Roseweir and Millar, 2009). Those reproductive neuroendocrine signaling pathways are evolutionarily highly conserved in mammals and non-mammalian vertebrates. However, mechanism of reproductive control in non-mammalian vertebrates is not always the same as in mammalian models (Zohar et al., 2010). For example, in teleost fish, the pituitary gland is directly innervated by neurosecretory fibers and lacka hypothalamo-pituitary portal system of the median eminence (Peter et al., 1990). Many teleost species possess at least two or three GnRH types (GnRH1, GnRH2, and GnRH3) (White et al., 1995) or multiple GnRH neuronal populations in the brain (Parhar, 2002). Recent studies have revealed the presence of two types of kisspeptin encoding genes (*kiss1* and *kiss2*) and two forms of kisspeptin receptor genes (*kissr1* and *kissr2*) in teleosts (Lee et al., 2009; Akazome et al., 2010; Um et al., 2010; Tena-Sempere et al., 2012; Gopurappilly et al., 2013). The multiplicity of neuroendocrine signaling pathways in teleosts are probably due to a gene duplication event (Lethimonier et al., 2004; Um et al., 2010), but several evidences have suggested their unique roles and functional significance in the variety of reproductive strategies in teleosts (Peter et al., 1990; White et al., 1995; Parhar, 2002; Lethimonier et al., 2004; Um et al., 2010; Zohar et al., 2010).

In vertebrates, the HPG axis is regulated by a variety of internal and external factors. For example, one of the endogenous key factors controlling reproductive processes are sex steroids feedback mechanism exerted by the gonads to the hypothalamus and pituitary (Fink, 1979). In addition to gonadal steroids, several factors such as stress, nutrition, and neurotransmitters are involved in the control of the HPG axis, in particular modulation of gonadotropin release (Gallo, 1980; Genazzani et al., 2000; Zohar et al., 2010). Neurotransmitters such as monoamine, amino acids and peptides are involved in the neuroendocrine control of reproduction (Gallo, 1980; Nock and Feder, 1982). In mammals, serotonin (5-hydroxytryptamine), a monoamine neurotransmitter is involved in a wide range of reproductive functions such as GnRH secretion, gonadotropin release, gonadal maturation and socio-sexual behaviors. On the other hand, serotonin system can be modulated by reproductive factors. In mammals, ovarian steroids such as progesterone and estrogen regulates the content of serotonin in the brain (Pecins-Thompson et al., 1996). In several mammalian species, serotonergic neurons are colocalized with estrogen receptor beta (Gundlah et al., 2001, 2005). These results indicate that serotonin and reproductive endocrine signaling pathways are closely associated. The functional interactions between serotonin and reproductive functions have also been demonstrated in teleosts (Somoza et al., 1988; Khan and Thomas, 1992). However, the serotonin system in teleost may play a unique role in the control of reproduction because of the variety of neuroendocrine signaling. This review summarizes the functional significance of serotonin in the teleosts reproduction.

## Serotonin system in teleost

### Organization of serotonin system

The organization of serotonin in the central nervous system is evolutionarily well conserved in the vertebrates (Lillesaar, 2011). In the brain of teleosts, three major serotonergic neural groups exist: (i) pretectal population, (ii) posterior tuberculum/hypothalamic populations, and (iii) raphe populations (Kah and Chambolle, 1983; Ekström and Van Veen, 1984; Frankenhuis-van den Heuvel and Nieuwenhuys, 1984; Margolis-Kazan et al., 1985; Johnston et al., 1990; Corio et al., 1991; Ekström et al., 1992; Batten et al., 1993; RodrıìGuez-Gómez et al., 2000; Lillesaar, 2011). In addition, serotonin-positive cells are also present in the pineal gland, area postrema, medulla oblongata and spinal cord in the brain of teleosts (Lillesaar, 2011). In teleost, serotonergic fibers from the brain directly project to the pituitary (Kah and Chambolle, 1983; Corio et al., 1991; Khan and Thomas, 1993; RodrıìGuez-Gómez et al., 2000). In some teleosts species, serotonin-immunoreactive cells also present in the pituitary (Kah and Chambolle, 1983; Ekström and Van Veen, 1984; Margolis-Kazan et al., 1985; RodrıìGuez-Gómez et al., 2000).

In mammals, serotonin is synthesized from the essential amino acid, L-tryptophan with help of catalysis by two enzymes: tryptophan hydroxylase (TPH) and amino acid decarboxylase (Fitzpatrick, 1999), whereas knowledge about mechanism of the control of brain serotonin synthesis in teleosts is still limited (Höglund et al., 2005). However, teleosts fish also preserve the molecules that are involved in homeostasis of serotonin such as TPH, serotonin transporter (SERT), which reuptakes serotonin into the presynaptic serotonergic nerve terminals to recycle serotonin (Murphy et al., 1998), and monoamine oxidase (MAO), the enzyme for degradation of serotonin (Bortolato et al., 2010).

**Figure 1:**
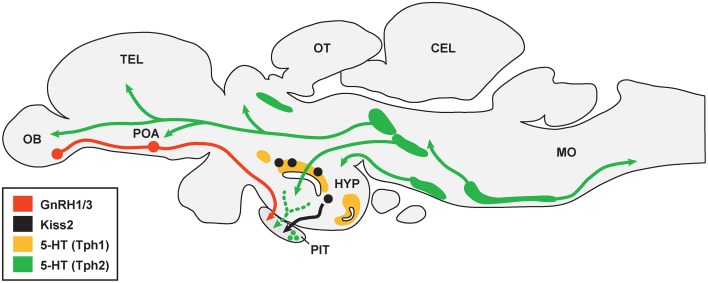
**Schematic drawing illustrating association between serotonergic cell populations with GnRH and kisspeptin neurons in the brain of teleosts**. There are multiple serotonergic (5-HT) cell populations that express either Tph1 (area shaded with *yellow*) or Tph2 (area shaded with *green*). 5-HT fibers may project to gonadotropin-releasing hormone (GnRH1 and GnRH3) neurons (shown in *red*) in the olfactory bulb (OB) and preoptic area (POA), while it is unknown whether 5-HT fibers are directly associated with kisspeptin (Kiss2) neurons (*black*) in the hypothalamus (HYP). 5-HT fibers and cells are also present in the pituitary (PIT), which may associate with GnRH and Kiss2 fibers in the pituitary. TEL, telencephalon; OT, optic tectum; CEL, cerebellum; MO, medulla oblongata. The organization of serotonergic projections were adopted from Lillesaar (2011) and Gaspar and Lillesaar (2012).

Most teleosts have two TPH genes (*tph1* and *tph2*), two SERT genes (*slc6a4a* and *slc6a4b*) but only one type of MAO gene (*mao*) (Chen et al., 1994; Setini et al., 2005; Norton et al., 2008; Rahman and Thomas, 2009). In some teleosts, such as zebrafish, stickleback and medaka, there are three genes (*tph1a*, *tph1b*, and *tph2*) encoding TPH (Lillesaar, 2011). In the brain of zebrafish, *tph1a* is present in the posterior tuberculum and hypothalamus, and also in the pineal organ, in amacrine cells of the retina, and *tph1b* is transiently expressed in a preoptic cell cluster during late embryonic stages (Bellipanni et al., 2002), and *tph2* is mainly expressed in serotonergic neurons of the raphe nuclei (superior raphe and inferior raphe) (Lillesaar, 2011) (Figure 1). In some teleosts, TPH is expressed in the pituitary (Boularand et al., 1998; Rahman and Thomas, 2009), indicating that serotonin may be locally produced in the pituitary. In the zebrafish, *slc6a4a* is expressed in the superior raphe and pretectal diencephalic cluster, and *slc6a4b* is seen only in the paraventricular organ and caudal zone of periventricular hypothalamus (Wang et al., 2006; Norton et al., 2008). In the serotonergic raphe nuclei, serotonergic neurons in the superior raphe project to the forebrain and midbrain, and the serotonergic cells in the inferior raphe project to hindbrain-spinal cord region in the teleosts brain (Lillesaar, 2011).

### Serotonin receptors

In teleosts, serotonin receptors have been identified and characterized in several species such as zebrafish, European flounder (*Platichthys flesus*), Gulf toadfish (*Opsanus beta*), and puffer fish (Yamaguchi and Brenner, 1997; Lu et al., 2007; Best and Alderton, 2008; Mager et al., 2012). Additionally, *in silico* analysis have predicted gene sequences encoding serotonin receptors in several other species such as the tilapia (*Oreochromis niloticus*), cichlid fish (*Haplochromis burtoni*), southern platyfish (*Xiphophorus maculatus*), and rainbow trout (*Oncorhynchus mykiss*). In the zebrafish, three serotonin receptors subtypes (5-HT1, 5-HT2, and 5-HT7) have been identified, among which three subgroups of 5-HT1 (5-HT1aa, 5-HT1ab, 5-HT1bd) and two subgroups of 5-HT2 (5-HT2A and 5-HT2C) have been identified (Norton et al., 2008; Schneider et al., 2012). In the brain of zebrafish, 5-HTr1aa and 5-HTr1ab are mainly expressed in the preoptic area and hypothalamus, and 5-HTr1bd is expressed in the hypothalamus (Norton et al., 2008). In the Gulf toadfish, 5-HT2A is widely expressed in the brain including the telencephalon, midbrain, cerebellum, hindbrain and in the pituitary (Mager et al., 2012). In the zebrafish, 5-HT2C is expressed in the telencephalon, diencephalon, rhombencephalon, and spinal cord (Schneider et al., 2012).

Serotonin receptors are also expressed in peripheral tissues including gonadal tissues in teleosts. In the zebrafish, 5-HT2C receptor gene is expressed in the ovary (Schneider et al., 2012). In the toadfish, 5-HT2A is expressed in the ovary and testes (Mager et al., 2012).

## Serotonin in teleost reproduction

### GnRH release

Serotonin modulates fish reproductive function via multiple pathways including through central (preoptic-hypothalamic area and pituitary) and peripheral (gonads) actions. In the hypothalamus, GnRH neurons play major role in the control of vertebrate reproduction. Immunohistochemical study in the Atlantic croaker have demonstrated close association of serotonin fibers with olfactory bulbular and hypothalamic GnRH neurons (Khan and Thomas, 1993). However, in the Atlantic croaker, central administration of serotonin has no effect on preoptic GnRH1 mRNA levels (Thomas et al., 2007), indicating that serotonin may stimulate GnRH release but not synthesis. Indeed, serotonin stimulates GnRH release from the hypothalamus of the seabream and goldfish (Yu et al., 1991; Senthilkumaran et al., 2001). In the zebrafish, expression of serotonin receptors are seen in several brain regions containing GnRH neurons (Norton et al., 2008), which suggests possible co-expression of serotonin receptors in GnRH neurons as in mammals (Bhattarai et al., 2013).

Kisspeptin, a ligand for G-protein coupled receptor GPR54, has recently emerged as a key player for GnRH release (Tena-Sempere, 2006; Gopurappilly et al., 2013). However, no report has described the involvement of serotonin in the regulation of the kisspeptin system in any vertebrates to date.

### Gonadotropin release

In Atlantic croaker increasing serotonin concentrations are associated with levels of gonadotropin release from the pituitary (Khan and Thomas, 1994). In several teleost species, serotonin stimulates release of gonadotropin *in vivo* and *in vitro* (Somoza et al., 1988; Somoza and Peter, 1991; Khan and Thomas, 1992). *In vitro* and *in vivo* studies in teleosts have shown the involvement of 5-HT1 or 5-HT1 receptor subtypes in stimulating gonadotropin secretion (Somoza and Peter, 1991; Khan and Thomas, 1994; Wong et al., 1998). These studies suggest that serotonin plays a prominent role in gonadotropin secretion in teleosts as demonstrated in mammals.

In the Atlantic croaker, serotonin combination with GnRH stimulates LH secretion (Wong et al., 1998). In the goldfish, serotonin stimulates release of GnRH from the cultured brain preoptic-anterior hypothalamic region and pituitary fragments (Yu et al., 1991). However, a recent *in vivo* study in Prussian carp (*Carassius gibelio* Bloch) demonstrated that serotonin alone had no influence on the spontaneous LH release, but the additive effects of serotonin was observed when GnRH analog was co-administered (Sokolowska-Mikolajczyk et al., 2015). These observations indicate functional interaction between serotonin and GnRH system in teleosts. However, an *in vitro* study in the red seabream demonstrated that serotonin stimulates the release of GnRH from the hypothalamus but not from the pituitary of immature fish (Senthilkumaran et al., 2001). Therefore, in teleosts, the mode of action of serotonin on gonadotropin release could be changed reproductive-stage dependently. Additionally, serotonin is also known to modulate growth hormone (GH) release in goldfish (Somoza and Peter, 1991; Wong et al., 1998). In the goldfish, GnRH-stimulated GH secretion is interfered by serotonin with PKC and Ca^2+^ signaling pathways in pituitary cells (Yu et al., 2008). Those signaling pathways could also be involved in GnRH-primed gonadotropin secretion in teleosts.

### Gonadal maturation

In addition to its central action on the reproductive axis, serotonin directly acts on gonads. In the Gulf killifish (*Fundulus grandis*), 10 days of daily injection of serotonin precursor with dopamine precursor increases gonadosomatic index in male (Emata et al., 1985). An *in vitro* study in the Japanese medaka (*Oryzias latipes*) has shown stimulatory effect of serotonin on oocyte maturation in a dose-dependent manner, which is modulated via stimulation of the synthesis of estrogen and the maturation-inducing steroids (MIS: 17α,20β-dihydroxy-4-pregnen-3-one) by the granulosa cells (Iwamatsu et al., 1993) On the contrary, in the mummichog (*Fundulus heteroclitus*), serotonin inhibits oocyte maturation, especially oocyte meiosis (Cerdá et al., 1995, 1997, 1998).

Although the expression of serotonin receptors in the testis has not been reported in teleosts, in freshwater catfish (*Channa punctatus* Bloch), MAO activity has been noted in the testis (Katti and Sathyanesan, 1986), and MOA activity and serotonin contents in testis represents correlative changes with testicular maturation (Joshi and Sathyanesan, 1980). These results suggest that locally produced serotonin may participate in testicular maturation.

### Social and reproductive behaviors

The role of serotonin in social behavior has been well demonstrated in fish (Winberg and Nilsson, 1993), while no report has demonstrated the involvement of serotonin in sexual behavior. As social status and reproductive activity are closely related, alteration of serotonin during different social status may directly influence reproductive activities. In teleosts fish, serotonin plays primary inhibitory role in aggressive behavior (Munro, 1986; Adams et al., 1996; Winberg et al., 2001; Perreault et al., 2003). In the fighting fish *Betta splendens*, serotonin decreases aggression via 5-HT1A receptors (Clotfelter et al., 2007). On the contrary, higher levels of serotonin metabolite are found in the brain of subordinate compared with dominant fish (Winberg and Lepage, 1998; Lorenzi et al., 2009). In a cichlid fish *Astatotilapia burtoni*, subordinate males have higher serotonergic turnover and higher expression of two serotonin receptor genes (5-HT1A and 2A) in the telencephalon (Loveland et al., 2014), indicating a correlation between social status and the serotonin system. In the Arctic charr (*Salvelinus alpinus L*.), higher brain serotonergic levels and activity is socially induced in subordinates (Winberg et al., 1991, 1992).

## Modulation of serotonin activity

### Gonadal steroids

In teleosts, serotonin levels in the brain and pituitary are modulated by reproductive cycles and gonadal steroids (Subhedar et al., 1997; Hernandez-Rauda and Aldegunde, 2002b). In the tilapia, estrogen alters the brain serotonin content during the early brain development stage, which is mediated by decreasing TPH activity and increasing MAO activity (Tsai and Wang, 1999). In the adult male marine yellow snapper (*Lutjanus argentiventris*), serotonin levels in the telencephalon reach the peak during the prespawning period, and are lowest during the spawning period (Hernandez-Rauda and Aldegunde, 2002a). Furthermore, blocking serotonin synthesis alters brain aromatase activity during the critical period of sexual differentiation in the tilapia (Tsai et al., 2000), suggesting possible involvement of serotonin in brain sex determination.

### Endocrine disruptors

Endocrine disruptors such as polyaromatic hydrocarbons (PAHs) and polychlorinated biphenyls (PCBs) can modulate serotonergic activity (Stephanou et al., 1998; Gesto et al., 2006; Clotfelter et al., 2010; Rahman et al., 2011). Some of these endocrine disruptors have a significant influence on fish reproductive function through the serotonin system. For example, PAHs such as naphthalene and benzo[α]pyrene disrupt the reproductive axis in teleosts (Hose et al., 1981; Yarahmadi et al., 2013). PCB inhibits serotonergic and TPH activity as well as disrupts GnRH and gonadotropin secretion in the Atlantic croaker (Khan and Thomas, 2000, 2006). Similarly, para-chlorophenylalanine (PCPA) reduces hypothalamic serotonin levels and impairs GnRH and LH secretion in the Atlantic croaker (Khan and Thomas, 2001). These results suggest that the serotonin system is one of the major targets for neuroendocrine disruption, which may lead to inhibition of reproductive functions.

### Environmental and social factors

In teleosts, the brain serotonergic activity displays diurnal or seasonal variations (Khan and Joy, 1988; Senthilkumaran and Joy, 1993), which may have significant effects on the reproductive functions. In teleosts, serotonin concentrations in the brain are higher in the morning than evening (Fingerman, 1976; Khan and Joy, 1988). In the *Channa punctatus*, there are diurnal variations in the serotonin content (Khan and Joy, 1988) as well as MAO activity in the hypothalamus (Khan and Joy, 1987, 1988), suggesting diurnal variation of the hypothalamic serotonin levels. Seasonal variation of hypothalamic serotonin content has also been noted in the catfish, *Heteropneustes fossilis* (Senthilkumaran and Joy, 1994). These seasonal changes in serotonin levels could also be due to environmental factors such as water temperature and photoperiod. In the tilapia, the hypothalamic serotonin content is lower in fish exposed to higher water temperature than those in lower temperature group (Tsai and Wang, 1997). In contrast, expression of serotonin receptors (5-HT1A and 1D) in the brain are increased by low temperature in the tilapia during the sexual differentiation (Wang and Tsai, 2006). In several fish species, photoperiods alter hypothalamic serotonin content and turnover (Olcese et al., 1980; Senthilkumaran and Joy, 1994), which can be modulated by melatonin levels (Joy and Khan, 1991). In the goldfish, pinealectomy and melatonin administration have a significant effect on hypothalamic serotonin content and serotonergic activity (Olcese et al., 1981). These results indicate environmental factors may influence reproductive functions via diurnal and seasonal change of serotonin activity.

In the protogynous fish, Hawaiian saddleback wrasse (*Thalassoma duperrey*), serotonin inhibits both initiation and completion of sex reversal (Larson et al., 2003a). Furthermore, in the same fish, serotonin levels in the brain are altered by socially induced sex reversal, which could be associated with territorial acquisition (Larson et al., 2003b). These results suggest that serotonin is also regulated by social behaviors.

### Selective serotonin reuptake inhibitor (SSRI)

Selective serotonin reuptake inhibitors (SSRIs) are widely used as antidepressants in the treatment of major depressive disorder and anxiety disorders (Lesch, 2001; Homberg et al., 2010). SSRIs have been detected as pharmaceutical contaminants in surface waters and sewage effluents (Kreke and Dietrich, 2008; Oakes et al., 2010) as well as in fish brain tissue (Schultz et al., 2010) owing to their widespread and increasing rates of administration. SSRIs block the presynaptic SERT and prevent the clearance of synaptic serotonin, which causes an elevation of extracellular serotonin concentrations (Tollefson and Rosenbaum, 1995). Chronic exposure to SSRIs cause significant decrease of serotonin content in the fish brain (Gaworecki and Klaine, 2008; Winder et al., 2009; Bisesi et al., 2014), which can influence the neuroendocrine control of reproductive function. Among the SSRIs, fluoxetine (also known as PROZAC) has been widely used to investigate the serotonergic modulation of the teleosts endocrine system (Somoza and Peter, 1991; Kreke and Dietrich, 2008). In female fish, fluoxetine treatment significantly reduces egg production and ovarian levels of estrogen, and gene expression levels of aromatase, FSH- and LH-receptors (Lister et al., 2009; Forsatkar et al., 2014). Conversely, fluoxetine has stimulatory effects on GnRH and LH release in some fish species (Somoza et al., 1988; Yu et al., 1991).

SSRIs also have influence on not only endocrine system, but also behaviors. In male fathead minnows (*Pimephales promelas*), exposure to sertraline, a SSRI decreases shelter-seeking behavior, suggesting that sertraline elicits an anxiolytic effect (Valenti et al., 2012). In hybrid striped bass (Morone saxatilis × M. chrysops), fluoxetine exposures decrease in ability of fish to capture prey (Gaworecki and Klaine, 2008). In the bluehead wrasse (*Thalassoma bifasciatum*), fluoxetine treatment decreases territorial aggression. However, in male *B. splendens*, there was no effect of chronic intramuscular injections of fluoxetine on aggressive behavior (Clotfelter et al., 2007). These observations suggest that environmental SSRIs may have significant impact on reproductive capability of fish via behavioral disruption.

A variety of influences of SSRIs on fish reproduction could be due to different doses, administrations, duration of SSRI treatments and physiological, reproductive status and sex of fish treated and species differences (Sumpter et al., 2014). However, it is still unclear how SSRIs act on the HPG axis via the serotonin system. In addition, most antidepressant drugs are specifically designed for humans (mammals), but not for fish. Therefore, the effects of these drugs may not be specific in teleosts. In fish, SSRIs are suggested to interact with and inhibit some P450 isozymes that are responsible for steroid metabolism (Kreke and Dietrich, 2008), which might have effect on the reproductive neuroendocrine control.

## Summary

**Figure 2:**
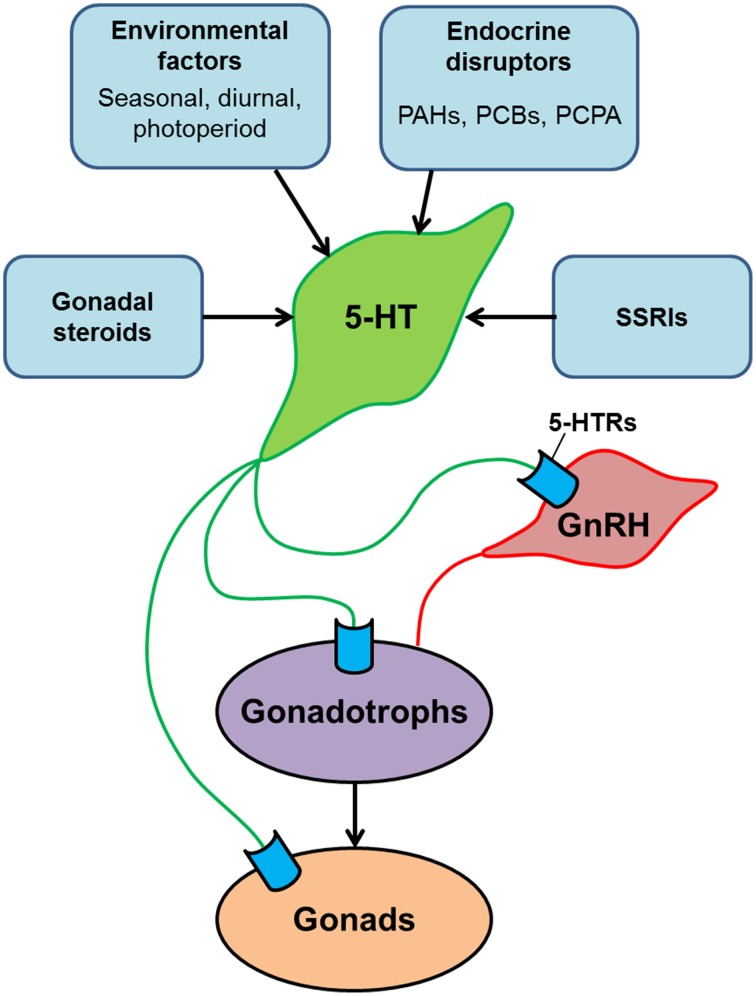
**Schematic model illustrating the serotonergic action on the hypothalamus-pituitary-gonadal axis of teleosts**. Serotonin (5-HT) modulates the reproductive system at multiple levels: the hypothalamus (via GnRH neurons), the pituitary (via gonadotrophs) and the gonads. 5-HT system is modulated by several factors such as gonadal steroids, environmental factors and social cues. In addition, central 5-HT system is also influenced by chemical substances such as endocrine disrupters and selective serotonin reuptake inhibitors (SSRIs), which exist in surface waters and sewage effluents as contaminants. Exposure of fish to those chemical substances may have significant impacts on reproductive functions.

Serotonin is one of the classic neurotransmitter and the structure of its related molecules such as TPH and SERT, and their brain organization are highly conserved in mammalian and non-mammalian vertebrates, suggesting functional conservation of the role of serotonin system in vertebrate reproduction. Several physiological studies have demonstrated the role of serotonin in a variety of reproductive functions including the control of GnRH release, LH release, gonadal maturation, and socio-sexual behaviors in teleosts (Figure 2). However, the serotonin system in teleost may also play unique role in the control of reproduction as the mechanism of reproductive control in teleosts is not always the same as in the mammalian models (Xiong et al., 1994; Zohar et al., 2010). For example, in some fish, there are serotoninergic cell populations in the hypothalamus and in the pituitary, which indicates the presence of multiple pathways of gonadotropin control by the serotonin system. In fish, the serotonin system is also regulated by natural environmental factors as well as chemical substances. In particular, SSRIs are commonly detected as pharmaceutical contaminants in the natural environment (Brooks et al., 2005; Corcoran et al., 2010). Several research articles demonstrate that acute and chronic exposure to SSRIs induces a variety of change in physiological and behavioral parameters in fish. However, environmental SSRIs could act on fish reproductive system via multiple pathways, the detail mechanisms underlying the effect of SSRIs on fish serotonin system and reproductive neuroendocrine system need to be examined to evaluate the potential influence of the SSRIs on fish reproductive functions.

### Conflict of interest statement

The authors declare that the research was conducted in the absence of any commercial or financial relationships that could be construed as a potential conflict of interest.
